# A Novel Rhabdovirus Associated with Acute Hemorrhagic Fever in Central Africa

**DOI:** 10.1371/journal.ppat.1002924

**Published:** 2012-09-27

**Authors:** Gilda Grard, Joseph N. Fair, Deanna Lee, Elizabeth Slikas, Imke Steffen, Jean-Jacques Muyembe, Taylor Sittler, Narayanan Veeraraghavan, J. Graham Ruby, Chunlin Wang, Maria Makuwa, Prime Mulembakani, Robert B. Tesh, Jonna Mazet, Anne W. Rimoin, Travis Taylor, Bradley S. Schneider, Graham Simmons, Eric Delwart, Nathan D. Wolfe, Charles Y. Chiu, Eric M. Leroy

**Affiliations:** 1 Viral Emergent Diseases unit, Centre International de Recherches Médicales de Franceville, Franceville, Gabon; 2 MIVEGEC, UMR (IRD 224 - CNRS 5290 - UM1 - UM2), Institut de Recherche pour le Développement, Montpellier, France; 3 Global Viral Forecasting, Incorporated, San Francisco, California, United States of America; 4 Department of Laboratory Medicine, University of California, San Francisco, California, United States of America; 5 UCSF-Abbott Viral Diagnostics and Discovery Center, San Francisco, California, United States of America; 6 Blood Systems Research Institute, San Francisco, California, United States of America; 7 Institut National de Recherche Biomédicale, Kinshasa, Democratic Republic of the Congo; 8 Howard Hughes Medical Institute, Chevy Chase, Maryland, United States of America; 9 Department of Biochemistry, University of California, San Francisco, California, United States of America; 10 Department of Biochemistry, Stanford University, Stanford, California, United States of America; 11 Department of Pathology, University of Texas Medical Branch, Galveston, Texas, United States of America; 12 Department of Epidemiology, University of California at Davis, Davis, California, United States of America; 13 Department of Epidemiology, University of California at Los Angeles, Los Angeles, California, United States of America; 14 Department of Medicine, Division of Infectious Diseases, University of California, San Francisco, San Francisco, California, United States of America; Washington University, United States of America

## Abstract

Deep sequencing was used to discover a novel rhabdovirus (Bas-Congo virus, or BASV) associated with a 2009 outbreak of 3 human cases of acute hemorrhagic fever in Mangala village, Democratic Republic of Congo (DRC), Africa. The cases, presenting over a 3-week period, were characterized by abrupt disease onset, high fever, mucosal hemorrhage, and, in two patients, death within 3 days. BASV was detected in an acute serum sample from the lone survivor at a concentration of 1.09×10^6^ RNA copies/mL, and 98.2% of the genome was subsequently *de novo* assembled from ∼140 million sequence reads. Phylogenetic analysis revealed that BASV is highly divergent and shares less than 34% amino acid identity with any other rhabdovirus. High convalescent neutralizing antibody titers of >1∶1000 were detected in the survivor and an asymptomatic nurse directly caring for him, both of whom were health care workers, suggesting the potential for human-to-human transmission of BASV. The natural animal reservoir host or arthropod vector and precise mode of transmission for the virus remain unclear. BASV is an emerging human pathogen associated with acute hemorrhagic fever in Africa.

## Author Summary

We used deep sequencing, a method for generating millions of DNA sequence reads from clinical samples, to discover a novel rhabdovirus (Bas-Congo virus, or BASV) associated with a 2009 outbreak of 3 human cases of acute hemorrhagic fever in Mangala village, Democratic Republic of Congo (DRC), Africa. The cases, presenting over a 3-week period, were characterized by abrupt disease onset, high fever, bloody vomiting and diarrhea, and, in two patients, death within 3 days. BASV was present in the blood of the lone survivor at a concentration of over a million copies per milliliter. The genome of BASV, assembled from over 140 million sequence reads, reveals that it is very different from any other rhabdovirus. The lone survivor and a nurse caring for him (with no symptoms), both health care workers, were found to have high levels of antibodies to BASV, indicating that they both had been infected by the virus. Although the source of the virus remains unclear, our study findings suggest that BASV may be spread by human-to-human contact and is an emerging pathogen associated with acute hemorrhagic fever in Africa.

## Introduction

**Figure 1:**
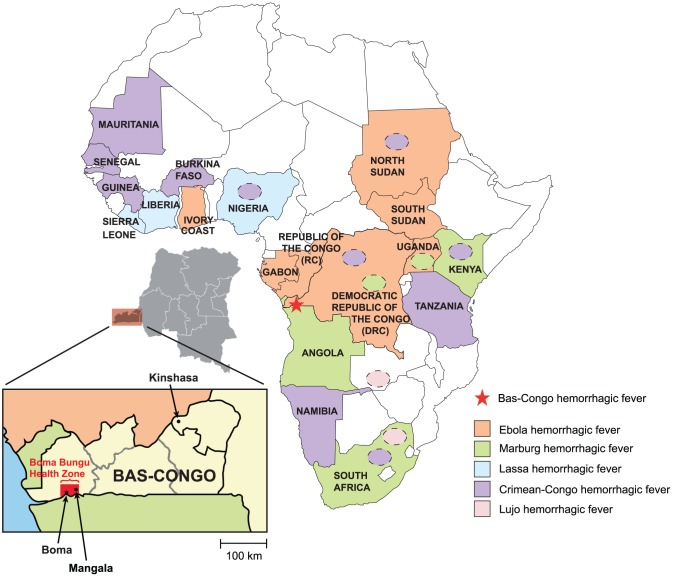
Map of Africa showing countries that are affected by viral hemorrhagic fever (VHF) outbreaks. Ebola VHF is pictured in orange, Marburg VHF in green, Crimean-Congo HF in violet, Lujo VHF in pink, and Lassa VHF in blue. Yellow fever and dengue VHF, which exhibit a wide geographic distribution throughout Sub-Saharan Africa, are not shown. Mangala village, located in the Bas-Congo province in DRC, is represented by a red star.

Viral hemorrhagic fever (VHF) encompasses a group of diseases characterized by fever, malaise, bleeding abnormalities, and circulatory shock [1], [2], [3]. Quality research on these infections is hindered by the fact that they are sporadic and often occur in geographically remote and politically unstable regions of the developing world. Most VHF diseases are associated with a short incubation period (2–21 days), abrupt onset, rapid clinical course, and high mortality, placing VHF agents amongst the most virulent human pathogens [4]. All known VHFs are zoonoses, and to date have been attributed to only four families of enveloped, single-stranded RNA viruses – *Arenaviridae*, *Bunyaviridae*, *Filoviridae* and *Flaviviridae*. Viruses from these families have caused major deadly outbreaks on the African continent (Fig. 1). Lassa fever virus (*Arenaviridae*) causes an estimated 500,000 cases each year in West Africa [5]. Crimean-Congo hemorrhagic fever (CCHF) and Rift Valley Fever viruses (*Bunyaviridae*) are associated with outbreaks in West, South and East Africa [6]. Ebola and Marburg viruses (*Filoviridae*) have caused several sporadic human outbreaks with high mortality (50–90%) in Central Africa, where they have also decimated local great ape populations [7]. Yellow fever and dengue viruses (*Flaviviridae)* are widely distributed throughout Sub-Saharan Africa where they cause both endemic and sporadic epidemic diseases in human populations [8].

Rhabdoviruses are members of the family *Rhabdoviridae* and order *Mononegavirales* and are enveloped viruses with single-stranded, negative-sense RNA genomes [9]. Their genomes encode at least five core proteins in the following order: 3′-nucleoprotein (N), phosphoprotein (P), matrix protein (M), glycoprotein (G) and large protein, or RNA-dependent RNA polymerase (L)-5′ (N-P-M-G-L). Rhabdoviruses are currently divided into six genera, with the two genera *Ephemerovirus* and *Vesiculovirus*, together with about 130 unclassified viruses, forming the dimarhabdovirus supergroup (“dipteran mammal-associated rhabdovirus”) [10]. Notably, although rhabdoviruses span all continents and exhibit a wide host range, infecting plants, invertebrates, vertebrate animals, and humans, relatively few are known to cause human infections. Rabies virus (RABV) and related viruses from the *Lyssavirus* genus and Chandipura virus (CHPV) from the *Vesiculovirus* genus are known to cause acute encephalitis syndromes [11], [12]. Other viruses from the genus *Vesiculovirus* cause vesicular stomatitis (mucosal ulcers in the mouth) and “flu-like” syndromes in both cattle and humans [13].

Unbiased next-generation or “deep” DNA sequencing is an emerging method for the surveillance and discovery of pathogens in clinical samples [14]. Unlike polymerase chain reaction (PCR), deep sequencing does not rely on the use of target-specific primers. Thus, the technique is particularly useful for the identification of novel pathogens with high sequence divergence that would elude detection by conventional PCR assays. Deep sequencing has been used previously to discover a new hemorrhagic fever-associated arenavirus from southern Africa, Lujo virus [15], as well as a new polyomavirus in human Merkel cell carcinoma [16]. With the depth of sequence data now routinely extending to >100 million reads, *de novo* genome assembly of novel viruses directly from primary clinical samples is feasible, as demonstrated by assembly of the 2009 pandemic influenza H1N1 virus genome from a single patient's nasal swab without the use of a reference sequence [17]. Here we report the critical role of deep sequencing in the discovery of a novel rhabdovirus associated with a small outbreak of fulminant hemorrhagic fever in the remote village of Mangala, Bas-Congo province, Democratic Republic of Congo (DRC), between May 25 and June 14, 2009.

## Results

### Case Reports from an Acute Hemorrhagic Fever Outbreak

#### Patient 1

The first case was a 15-year-old boy who presented to the health center in Mangala village (Boma Bungu Health Zone) on May 25, 2009 with malaise, epistaxis (nose bleeding), conjunctival injection, gingival bleeding, hematemesis (vomiting with blood), and watery diarrhea with blood (Table 1). No fever or respiratory symptoms were noted. Hemorrhagic symptoms initially appeared on May 24, and the patient died 2 days later from sudden circulatory collapse. The patient lived in the Tshela neighborhood of Mangala village and attended the local public school. All close contacts were monitored for 21 days, and none developed any signs of illness.

**Table 1:** Demographics of and clinical symptoms developed in the three patients suspected to be infected by Bas-Congo virus (BASV).

	Patient 1	Patient 2	Patient 3
Sex	Male	Female	Male
**Age**	15	13	32
**Village**	Mangala	Mangala	Mangala
**Neighborhood**	Tshela	Tshela	Tshela
**Occupation**	Schoolboy	Schoolgirl	Nurse
**Disease onset**	May 24	June 4	June 13
**Time until death**	2 days	3 days	survived
**Fever (T>39°C)**	No	Yes	Yes
**Weakness**	No	No	Yes
**Malaise**	Yes	No	No
**Headache**	No	Yes	Yes
**Abdominal pain**	No	Yes	Yes
**Epistaxis (nose bleeding)**	Yes	Yes	Yes
**Ocular hemorrhage/conjunctival injection (eye bleeding)**	Yes	Yes	Yes
**Oral hemorrhage (mouth bleeding)**	Yes	Yes	Yes
**Hemorrhagic vomiting**	Yes	Yes	Yes
**Hemorrhagic diarrhea**	Yes	Yes	Yes

#### Patient 2

The second case was a 13-year-old girl. She attended the same public school as Patient 1 but was in a different class. She also lived in the Tshela neighborhood of Mangala village, about 50 meters from Patient 1′s house. They knew each other but had no known face-to-face contact during the previous weeks. This patient presented to the health center on June 5, 2009 with headache, fever, abdominal pain, epistaxis, conjunctival injection, mouth bleeding, hematemesis, and diarrhea with blood. She was examined by a nurse and received acetaminophen and dipyrone for fever and quinine for possible malaria. Symptoms appeared on June 4, and the patient died suddenly on June 7, three days after onset. None of her close contacts developed symptoms during the 21 days of monitoring after her death.

#### Patient 3

The third case was a male nurse aged 32 years working in the health center visited by Patients 1 and 2. His disease appeared suddenly on June 13, 2009 with epistaxis, ocular and oral hemorrhage, hematemesis, and diarrhea with blood. Two days after the onset of hemorrhagic symptoms, he developed fever, anorexia, headache, fatigue, and abdominal pain. He was transferred to the regional general hospital of Boma (Fig. 1), a city of about 200,000 inhabitants, where a serum sample was obtained on June 15, just prior to treatment with fluid resuscitation, blood transfusion, and empiric antibiotics. Laboratory tests for malaria, tuberculosis, dengue, and bacterial sepsis were negative, and the patient recovered spontaneously a few days later. All persons in Mangala and Boma who had contact with Patient 3 were monitored for 21 days, and none became ill. Patient 3, like the two other patients, lived in the Tshela neighborhood of Mangala village, about 50 meters from Patients 1 and 2. Importantly, patient 3 was directly involved in the care of Patients 1 and 2 when they presented to the health center with hemorrhagic symptoms.

No disease outbreaks had been reported in the past in Boma Bungu Health Zone with the exception of a cholera diarrheal outbreak in 2006, and, notably, no cases of hemorrhagic disease had previously been reported. In addition, although DRC is a country endemic for filovirus infection (Fig. 1), no outbreaks of Ebola or Marburg fever have ever been described in Bas-Congo province. No animal die-offs or other unusual events in association with these cases were noted.

### Initial Sample Collection and Diagnostic Testing

A cluster of three human cases of typical acute hemorrhagic fever occurred between May 25 and June 13, 2009 in Mangala village, located in a remote tropical forest region in Central Africa. Cases were characterized by abrupt disease onset, high fever of >39°C when present, overt hemorrhagic symptoms with epistaxis, conjunctival injection, mouth and gastrointestinal bleeding, followed by death within 3 days of symptom onset in two patients (Table 1). The first patient, who died <48 hours after presentation, exhibited hemorrhagic symptoms without a documented fever, and only the third adult patient recovered from his illness. All three patients lived within a 2500-m^2^ area in the same neighborhood of Mangala, a remote village in Bas-Congo province of DRC (Fig. 1). The first two patients died rapidly in Mangala village, and no blood samples were collected. A blood sample was collected from the third surviving patient three days after symptom onset and sent to Centre International de Recherches Médicales de Franceville (CIRMF) for etiological diagnosis. The sample tested negative by TaqMan real-time PCR assays for all viruses known to cause acute hemorrhagic fever in Africa (data not shown).

### Discovery and Genome Assembly of the BASV Rhabdovirus

**Figure 2:**
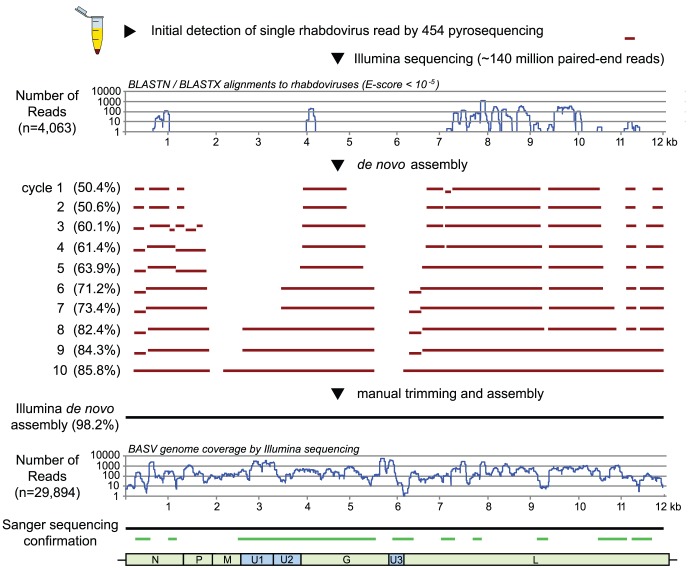
Deep sequencing and whole-genome *de novo* assembly of BASV. After initial discovery of BASV from a single 454 pyrosequencing read, 98.2% of the BASV genome was assembled *de novo* from >140 million paired-end Illumina reads. The horizontal lines (red) depict regions of the genome successfully assembled at the end of each cycle. PCR and Sanger sequencing were performed to confirm the assembly and genomic organization of BASV (green lines).

To identify a potential causative pathogen in the third surviving patient with unknown hemorrhagic fever, RNA extracts from the serum sample were analyzed using unbiased deep sequencing (Fig. 2). The initial Roche 454 pyrosequencing library yielded a total of 4,537 sequence reads, of which only a single 220 bp read (0.022%) aligned with any annotated viral protein sequence in GenBank. The translation product showed similarity to a segment of the L protein, or RNA-dependent RNA polymerase, from Tibrogargan and Coastal Plains rhabdoviruses, with 41% identity to Coastal Plains virus (GenBank ADG86364; BLASTx E-score of 2×10^−6^). This finding suggested the presence of a novel, highly divergent rhabdovirus in the patient's serum. Attempts to extend the initial sequence by primer walking or PCR using rhabdovirus consensus primers failed due to limited sample availability; thus, we resorted to ultra-deep sequencing on an Illumina HiSeq 2000. Out of the 140,164,344 reads generated from Illumina sequencing, 4,063 reads (0.0029%) had nucleotide or protein homology to rhabdoviruses with an E-score of <10^−5^. These reads were used as “seeds” for iterative *de novo* assembly, resulting in construction of an estimated 98.2% of the genome of the novel rhabdovirus. We provisionally named this rhabdovirus BASV, or Bas-Congo virus, referring to the province from which the outbreak originated.

The coverage of BASV achieved by deep sequencing was at least 10-fold across nearly the entire genome and included 29,894 reads out of ∼140 million (0.021%) (Fig. 2). The viral load in the patient's serum was 1.09×10^6^ RNA copies/mL by quantitative RT-PCR. The only moderately high titer is consistent with the fact that the sampled patient was a survivor of BASV infection and would thus be anticipated to have relatively lower viral titers in the blood, as also seen for survivors of Ebola virus infection [18].

Cultivation of the patient's serum in Vero, BHK, LLC-MK_2_ (rhesus monkey kidney), CCL-106 (rabbit kidney) and C6/36 (*Aedes albopictus* mosquito) cell cultures failed to show cytopathic effect, and serial quantitative BASV RT-PCR assays on primary and passaged cell culture supernatants turned negative. Subsequent electron microscopy of inoculated cell cultures was negative for viral particles. In addition, no illnesses or deaths occurred in suckling mice inoculated intracerebrally with the BASV-positive serum and observed over 14 days.

### Phylogenetic Analysis of BASV and Comparison with other Rhabdoviruses

**Figure 3:**
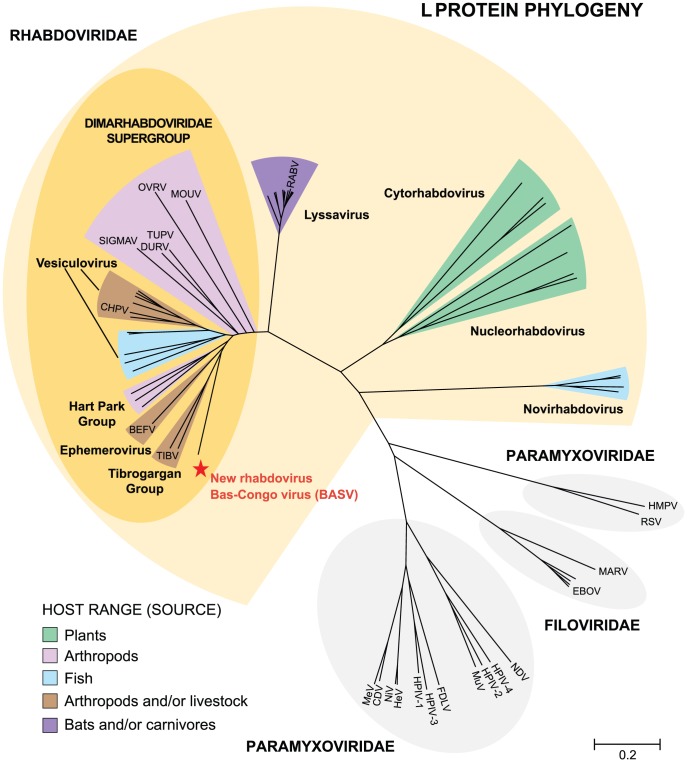
Phylogenetic analysis of the L proteins of BASV and other viruses in the order *Mononegavirales*. The host from which each virus was isolated is represented by a specific color. To generate the *Mononegavirales* (*Rhabdoviridae*, *Filoviridae* and *Paramyxoviridae*) phylogeny trees, all complete sequences of the large (L) protein, or RNA-dependent RNA polymerase (2000–2300 amino acids in length) were downloaded from GenBank. Abbreviations and accession numbers used for the phylogenetic analysis are provided in Methods.

**Figure 4:**
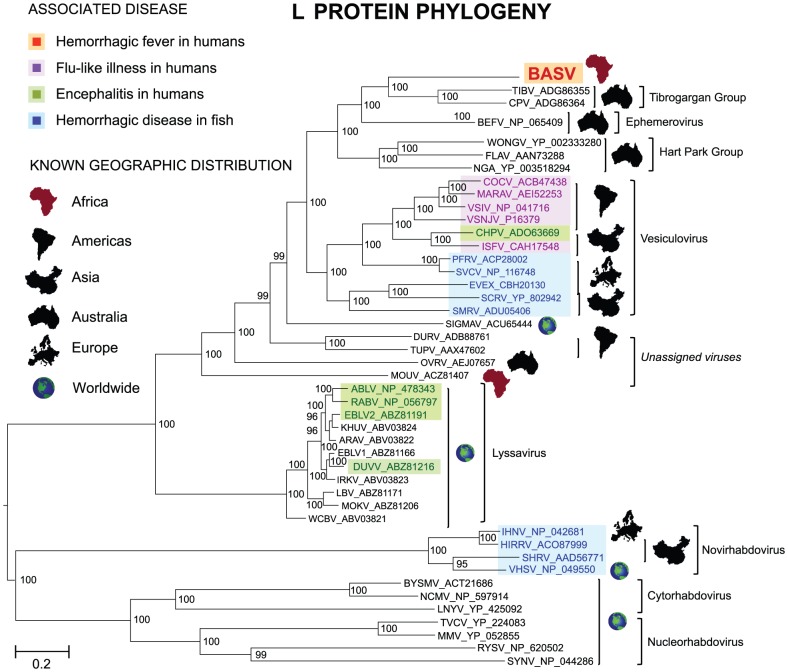
Phylogenetic analysis of the L proteins of BASV and other rhabdoviruses. The geographic distribution for each virus or group of viruses is indicated with a specific icon, while diseases associated with infection by certain rhabdoviruses are indicated by specific colors. Abbreviations and accession numbers used for the phylogenetic analysis are provided in Methods.

**Figure 5:**
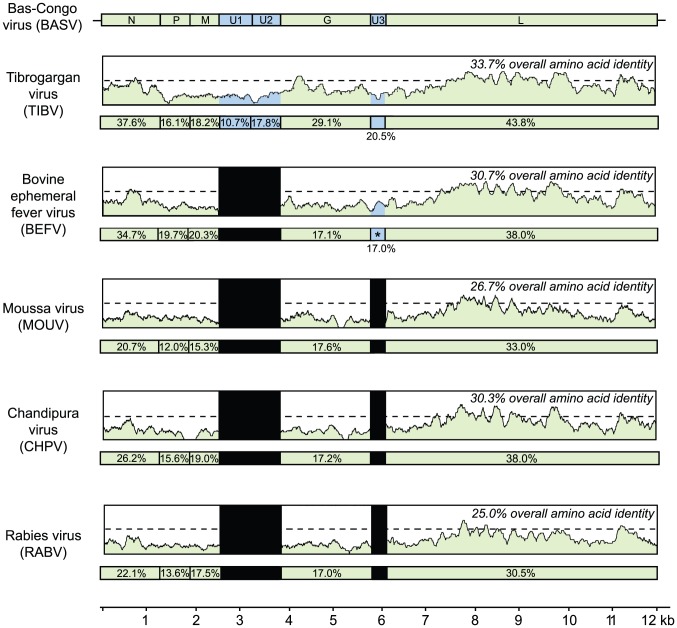
Schematic representation of the genome organization of BASV and its protein similarity plot compared to representative rhabdoviruses. The similarity plots are generated by aligning the concatenated rhabdovirus proteins and calculating scanning amino acid pairwise identities using a window size of 50 bp. The horizontal bar under each similarity plot shows the percent identity of the rhabdovirus protein relative to its corresponding protein in BASV. Genes coding for the 5 core rhabdovirus proteins are shown in green, while the accessory U1, U2, or U3 genes are shown in blue. Black bars correspond to accessory proteins which are not present in the genome. Note that BEFV contains 3 genes between G and L; only the alignment between the alpha-1 protein of BEFV and the U3 protein of BASV is shown (asterisk). The x-axis refers to the nucleotide position along the ∼12 kb genome of BASV.

Phylogenetic trees reveal that BASV belongs to the *dimarhabdoviridae* supergroup and is distantly related to members of the Tibrogargan group and the *Ephemerovirus* genus, although it clusters separately from other rhabdoviruses in an independent deeply rooted branch (Figs. 3 and 4; Fig. S1). Comparative analysis of the concatenated BASV proteins with representative dimarhabdoviruses reveals very low overall amino acid pairwise identity of 25.0 to 33.7%, depending on the virus (Fig. 5). Notably, BASV diverges significantly from either of the two main recognized human pathogens among rhabdoviruses, rabies virus or Chandipura virus.

The sequence divergence of BASV relative to other rhabdoviruses is also correlated with differences in genome structure (Fig. 5). The prototype genome organization of rhabdoviruses, found in lyssaviruses, is N-P-M-G-L. However, molecular analysis of novel rhabdoviruses has often revealed more complex genomes, with up to 10 additional open reading frames (ORF) located within an existing gene or interposed between the five core genes [19], [20], [21]. Rhabdoviruses from the Tibrogargan group (TIBV and CPV) share a distinctive genome structure with three additional genes, two between M and G (U1 and U2) and one between G and L (U3) [22]. Interestingly, BASV also has these three additional genes (U1–U3), confirming the phylogenetic relationship and overall structural similarity to the Tibrogargan group viruses. Based on their size, the U3 proteins of TIBV, CPV, and presumably BASV are candidate viroporins [22]. BASV is more distant structurally and phylogenetically from the Ephemero and Hart Park Group rhabdoviruses (Figs. 3 and 4), which do not contain U1 or U2 genes, but rather an additional two or three genes between G and L (including a putative U3 viroporin in BEFV referred to as the alpha-1 protein) (Fig. 5, asterisk). Moussa virus (MOUV), another rhabdovirus recently discovered in Africa (Fig. 4), does not contain any accessory genes but instead, shares the prototype N-P-M-G-L rhabdovirus structure [23].

### BASV Serological Testing of the Case Patient and Close Contacts

**Figure 6:**
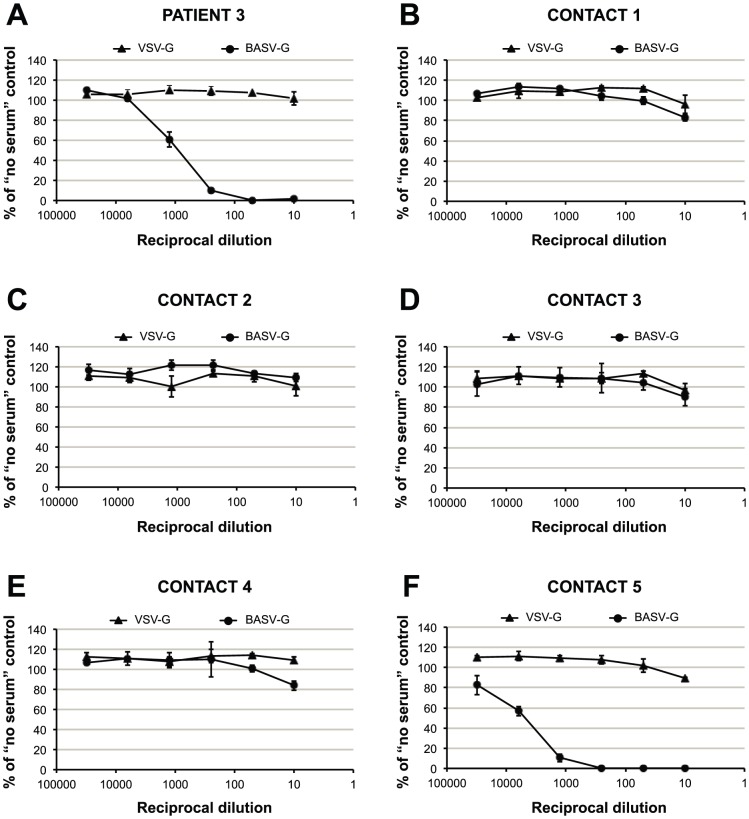
Detection of antibodies to BASV by serum neutralization of VSVΔG-GFP pseudotypes. Infectivities of VSVΔGFP pseudotypes bearing the glycoproteins of VSV or BASV, respectively, after incubation with 5-fold serial dilutions (1∶10, 1∶50, 1∶250, 1∶1,250, 1∶6,250, 1∶31,250) of sera from six individuals are depicted as percent of infectivity in the absence of serum. The six individuals tested include a patient with hemorrhagic fever (panel A, “Patient 3”), the nurse directly caring for him (panel F, “Contact 5”), and other health care workers in Mangala village (panels B–E). All data points represent the average of triplicate assays; error bars indicate standard deviations. Similar results were obtained in an independent experiment using murine leukemia virus (MLV)-based pseudotypes (data not shown).

To confirm that BASV is infectious to humans, convalescent sera were collected in early 2012 from surviving Patient 3 as well as five additional health care workers from Mangala identified as close contacts and tested in a blinded fashion for the presence of neutralizing antibodies to BASV (Fig. 6). Two of the six sera tested strongly positive with 50% protective doses between 1∶1,000 and 1∶5,000 (Figs. 6A and 6F). Moreover, the observed neutralization was highly specific for BASV-G, since no neutralization was observed with pseudoviruses harboring the vesicular stomatitis virus glycoprotein (VSV-G). One of the neutralizing sera had been collected from surviving Patient 3 (Fig. 6A, “Patient 3”), whereas the other serum sample, containing even higher titers, corresponded to an asymptomatic nurse directly caring for Patient 3 during his period of acute hemorrhagic illness (Fig. 6F, “Contact 5”). Specifically, Contact 5 was the primary health care provider to Patient 3 at the health center and during his transfer to the general hospital at Boma. All 6 individuals, including Patient 3, tested negative for BASV viremia by specific RT-PCR (data not shown).

### Epidemiological Screening for BASV in the DRC

**Figure 7:**
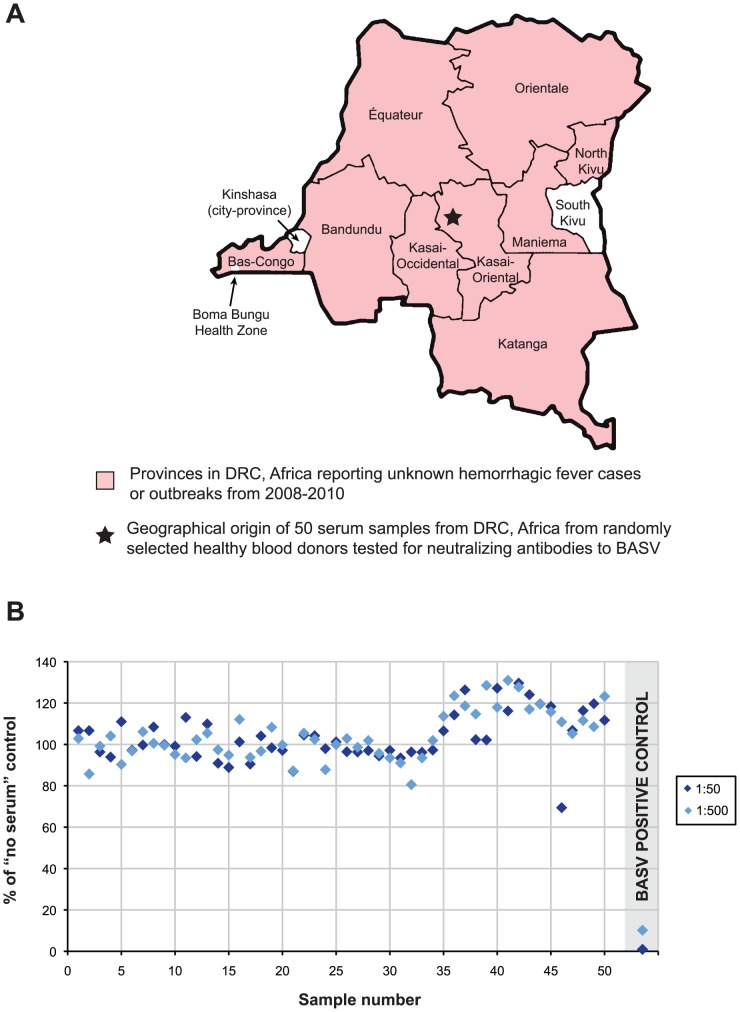
BASV Screening in DRC, Africa. (**A**) All 43 serum samples corresponding to unknown hemorrhagic fever cases or outbreaks in 2008–2010 from 9 provinces in DRC (pink) tested negative for BASV by PCR. (**B**) Sera from 50 donors in Kasai-Oriental province, DRC (Panel A, star) were tested for BASV-neutralizing antibodies. Sera at 1∶50 (dark blue) or 1∶500 dilution (light blue) were tested. Serum from the surviving Patient 3 was included as a positive control (grey shaded area). Data points represent an average of duplicate assays.

BASV was not detected by PCR in 43 serum samples from other unknown cases or outbreaks of hemorrhagic fever reported in the DRC from 2008–2010 (Fig. 7A, pink). Five of these 43 samples originated from the Bas-Congo outside of Mangala village and the Boma Bungu Health Zone. In total, the unknown hemorrhagic cases/outbreaks spanned 9 of the 11 provinces in the DRC, and all 43 samples also tested negative by PCR for the known hemorrhagic fever viruses circulating in Africa (data not shown). Fifty plasma samples collected from randomly selected blood donors in the Kasai-Oriental province of DRC (Fig. 7A, star; Table S2) were also screened and found to be negative for BASV-neutralizing antibodies (Fig. 7B).

## Discussion

Among more than 160 species of rhabdoviruses identified to date, fewer than 10 have been isolated from humans [24]. In addition, while human infection by rhabdoviruses has previously been associated with encephalitis, vesicular stomatitis, or “flu-like” illness, the discovery of BASV is the first time that a member of the *Rhabdovirus* family has been associated with hemorrhagic fever in humans with a fulminant disease course and high fatality rate. To our knowledge, this is also the first successful demonstration of *de novo* assembly of a novel, highly divergent viral genome in the absence of a reference sequence and directly from a primary clinical sample by unbiased deep sequencing.

Several lines of evidence implicate BASV in the hemorrhagic fever outbreak among the 3 patients in Mangala. First, this virus was the only credible viral pathogen detected in the blood of the lone survivor during his acute hemorrhagic illness by exhaustive deep sequencing of over 140 million reads. Analysis of the Illumina deep sequencing reads for the presence of other viral pathogens yielded only endogenous flora or confirmed laboratory contaminants (Table S1 and Fig. S2). Some enteric pathogens, such as E. coli O157:H7, Campylobacter, Shigella, and Salmonella, are diagnosed through fecal laboratory testing and not blood, and have been associated with hemorrhagic diarrhea [25]. However, these outbreaks are typically foodborne and associated with larger clusters and much greater numbers of clinical cases than reported here [26], [27], [28]. Furthermore, enteric diarrheal cases rarely present with systemic symptoms such as fever or generalized mucosal hemorrhage, with bleeding most often limited to the gastrointestinal tract, and overall mortality rates are generally low [26]. Thus, the clinical syndrome observed in 3 patients with hemorrhagic fever in the DRC, a region endemic for viral hemorrhagic fevers, is much more consistent with infection by a VHF disease agent. BASV is a plausible hemorrhagic fever candidate because it is a novel, highly divergent infectious virus, thus of unknown pathogenicity, and was detected at a titer of >1 million copies/mL in blood from an acutely ill individual. In addition, there is ample precedent for hemorrhagic disease from rhabdoviruses, as members of the genus *Novirhabdovirus* cause severe hemorrhagic septicemia in fresh and saltwater fish worldwide [29] (Fig. 4). The detection of BASV seropositivity in an asymptomatic close contact (Fig. 6) is not surprising given that up to 80% of patients infected with Lassa virus do not exhibit any hemorrhagic fever symptoms [30], [31].

Prior to the BASV outbreak, no hemorrhagic disease cases had been reported in Boma Bungu Health Zone. BASV was also not detected in 43 serum samples from unknown, filovirus-negative cases or outbreaks of hemorrhagic fever from 2008–2010 spanning 9 of the 11 provinces in the DRC (Fig. 7A). In addition, a serosurvey of 50 random blood donors from Kasai-Oriental province in central DRC was negative for prior exposure to BASV (Fig. 7B). Taken together, these data suggest that the virus may have emerged recently and locally from Boma Bungu in Bas-Congo, DRC.

We were unable to isolate BASV despite culturing the RNA-positive serum in a number of cell cultures and inoculation into suckling mice. One explanation for these negative findings may be that the virus inoculation titers of <50 µL were insufficient, although this is surprising given the concentration of >1 million copies per mL of BASV in blood from the lone survivor. A more likely explanation is viral inactivation resulting from the lack of adequate cold chain facilities in remote Boma Bungu. Viral RNA can often still be detected by RT-PCR in sera that is culture-negative [32]. In support of this premise, we have observed that the BASV-G/VSVΔG-GFP pseudotyped virus efficiently infects and replicates in a variety of insect and mammalian (including human) cell lines (Steffen, *et al.*, manuscript in preparation). In the absence of a positive culture, a “reverse genetics” approach to produce recombinant BASV particles, if successful, would greatly facilitate further study of the virus, as established previously for other rhabdoviruses such as VSV [33].

Based on our findings, some speculations on the origin of and routes of transmission for BASV can be made. All 3 patients became ill with acute hemorrhagic fever over a 3-week period within the same 2500-m^2^ area of Mangala village, suggesting that all 3 cases were infected with the same pathogen. Waterborne or airborne transmission would be expected to result in more numerous cases than the 3 reported. There were no reports of animal die-offs that would suggest potential exposures to infected wild animals or livestock. Taken together, these observations suggest that an unknown arthropod vector could be a plausible source of infection by BASV. This hypothesis is consistent with the phylogenetic and structural relationship of BASV to rhabdoviruses in the Tibrogargan group and *Ephemerovirus* genus, which are transmitted to cattle and buffalo by *Culicoides* biting midges [9]. In addition, the recent discovery of Moussa virus (MOUV), isolated from *Culex* mosquitoes in Cote d'Ivoire, Africa [23], implies the presence of hitherto unknown arthropod vectors for rhabdoviruses on the continent. Nevertheless, at present, we cannot exclude the possibility of other zoonotic sources for the virus or even nosocomial bloodborne transmission (as Patients 1 and 2 have not clearly been established to be BASV cases by serology or direct detection), and the natural reservoir and precise mode of transmission for BASV remain unknown. A community-based serosurvey in Boma Bungu and an investigation to track down potential arthropod or mammalian (e.g. rodents and bats) sources for BASV are currently underway.

**Figure 8:**
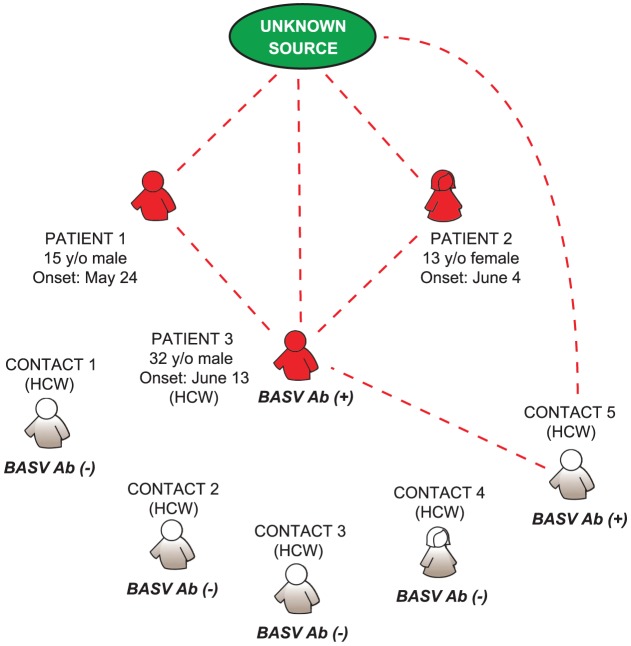
Proposed model for BASV transmission during the hemorrhagic fever outbreak in Mangala. Patients presenting with symptoms of acute hemorrhagic fever are depicted in red. Dashed red lines represent potential routes of BASV transmission. Contacts 1 through 5 are health care workers at the local health center in Mangala village. Abbreviations: HCW, health care worker; y/o, year-old; Ab, antibody.

Although we cannot exclude the possibility of independent arthropod-borne transmission events, our epidemiologic and serologic data do suggest the potential for limited human-to-human transmission of BASV. Patient 3, a nurse, had directly taken care of Patients 1 and 2 at the health center, and another nurse (Contact 5), who had taken care of Patient 3 (but not Patients 1 or 2) had serologic evidence of asymptomatic BASV infection. We present a hypothetical model for BASV transmission during the hemorrhagic fever outbreak in which the initial infection of two children in Mangala (Patients 1 and 2) was followed by successive human-to-human transmission events involving two healthcare workers (Patient 3 and Contact 5) (Fig. 8). This pattern of transmission from the community to health care workers is also commonly seen in association with outbreaks of Ebola and Crimean-Congo hemorrhagic fever [6], [34].

While rhabdoviruses are distributed worldwide, some authors have suggested that the *Rhabdoviridae* family probably originated from tropical regions of the Old or New World [9]. The discovery of BASV in Central Africa suggests that additional rhabdoviruses of clinical and public health importance likely await identification, especially in these poorly investigated geographic regions. Active epidemiological investigation and disease surveillance will be needed to fully ascertain the clinical and public health significance of BASV infection in humans, as well as to prepare for potentially larger human outbreaks from this newly discovered pathogen.

## Methods

### Ethics Statement

Written informed consent for publication of their case reports was obtained from the sole survivor of the hemorrhagic fever outbreak and the parents of the two deceased children. Written informed consent was obtained from the surviving patient and 5 of his close contacts for analysis of the serum samples reported in this study. Samples were analyzed under protocols approved by the institutional review boards of University of California, San Francisco, the University of Texas Medical Branch, and the National Institute of Biomedical Research (INRB) and CIRMF in Gabon, and the Institutional Animal Care and Use Committee (IACUC) of the University of Texas Medical Branch.

### Diagnostic Samples

No diagnostic samples were available from Patient 1 or Patient 2. Blood was collected in a red top serum tube from Patient 3 on June 16, during the acute phase, three days after hemorrhagic onset. The sample was transported at 4°C to the BSL-4 facility at CIRMF. Serum was obtained by centrifugation at 2300 rpm for 10 min. No other acute samples from Patient 3 were available. In January of 2012 (∼2.5 years after the outbreak), convalescent sera were collected from Patient 3 and close contacts (other workers at the health center) for BASV neutralization testing. Forty-three serum samples from other unknown hemorrhagic fever cases or outbreaks representing 9 of 11 provinces in the DRC were available for BASV PCR testing (Fig. 7A). Fifty available plasma samples from random blood donors (median age 27.5 years; age range 1–76 years) in Kasai Oriental province, DRC, were also tested for antibodies to BASV (Fig. 7A and B; Table S2).

### Nucleic Acid Extraction and Viral PCR Testing

RNA was extracted from 140 µl of serum using the QIAamp viral RNA mini kit (Qiagen). Taqman real-time reverse-transcription-PCR (RT-PCR) testing for known hemorrhagic fever viruses was performed using primers and probes specific for Marburg virus (MARV), all four species of Ebola virus (Zaire, ZEBOV; Sudan, SEBOV; Côte d'Ivoire, CIEBOV, and Bundibugyo, BEBOV), Crimean-Congo hemorrhagic fever virus (CCHFV), Yellow fever virus (YFV), Dengue virus (DENV), Rift Valley fever virus (RVFV) and Chikungunya virus (CHIKV) (available upon request).

### Discovery of the BASV Rhabdovirus by 454 Pyrosequencing

200 µL of serum sample were inactivated in 1 mL of TRIzol (Invitrogen), and nucleic acid extraction and purification were performed according to the manufacturer's instructions. Roche 454 pyrosequencing using randomly amplified cDNA libraries was performed as described previously [35]. Viral sequences were identified using BLASTn or BLASTx by comparison to the GenBank nonredundant nucleotide or protein database, respectively (E-score cutoff = 10^−5^).

### *De novo* Assembly of the BASV Genome by Illumina Sequencing

To recover additional BASV sequence, two sets of cDNA libraries were prepared from DNase-treated extracted RNA using a random PCR amplification method as described previously [36], or random hexamer priming according to the manufacturer's protocol (Illumina). The libraries were then pooled and sequenced on two lanes of an Illumina HiSeq 2000. Raw Illumina sequences consisting of 100 base pair (bp) paired-end reads were filtered to exclude low-complexity, homopolymeric, and low-quality sequences, and directly compared using BLASTn or BLASTx alignments to a library consisting of all rhabdovirus sequences in GenBank. The initial read obtained by 454 pyrosequencing as well as other reads aligning to rhabdoviruses were then inputted as “seeds” into the PRICE *de novo* assembler [37] (Fig. 2), with a criterion of at least 85% identity over 25-bp to merge two fragments. *De novo* assembly of the BASV genome was performed iteratively using PRICE and the Geneious software package (Biomatters) [38]. The near-complete whole genome sequence of the novel rhabdovirus (∼98.2% based on protein homology to other rhabdoviruses) was determined to at least 3× redundancy by *de novo* assembly as well as PCR and Sanger sequencing of low-coverage regions. Sanger sequencing was also performed to verify the accuracy of the assembly and confirm the genomic organization of BASV (Fig. 2).

### Deep Sequencing Analysis of the BASV Serum Sample for Other Pathogens

Rapid classification of the ∼140 million 100-bp paired-end Illumina reads was performed using a modified cloud computing-based computational analysis pipeline [17] (Veeraraghavan, Sittler, and Chiu, manuscript in preparation). Briefly, reads corresponding to human sequences were taxonomically classified using SOAP and BLAT software [39], [40]. Other reads were then identified using BLASTn or BLASTx by comparison to GenBank-derived reference databases (E-score cutoff = 10^−5^).

### PCR Quantitation of BASV Burden

To estimate the viral load in the patient's serum, we first designed a set of specific PCR primers for detection of BASV targeting the L protein, BASV-F (5′- CGCTGATGGTTTTTGACATGGAAGTCC-3′)/BASV-R (5′-TAAACTTCCTCTCTCCTCTAG-3′), for use in a SYBR-Green real-time quantitative RT-PCR assay. A standard curve for the assay was constructed as described previously [36]. The viral load in the patient's serum was determined by comparison to the standard curve.

### Structural Features and Phylogenetic Analysis

Predicted open reading frames (ORFs) in the BASV genome were identified with Geneious [38]. Multiple sequence (Figs. 3 and 4; Fig. S1) and pairwise (Fig. 5) alignments of BASV proteins relative to corresponding proteins from other rhabdoviruses were calculated using MAFFT (v6.0) with the E-INS-i option and at default settings [41]. To generate the phylogeny trees, all rhabdoviruses in GenBank were included as well as representative members of other families within the order *Mononegavirales*. Bayesian tree topologies were assessed with MrBayes V.32 software (20,000 sampled trees; 5,000 trees discarded as burn-in) [42]. Convergence was confirmed by the PSRF statistic in MrBayes, as well as by visual inspection of individual traces using TRACER from the BEAST software package [43]. Trees were visualized after midpoint rooting with FigTree V1.31 [43].

### Virus Cultivation in Cell Cultures or Suckling Mice

Initial attempts were made to culture the virus using a total of 200 µL of BASV-positive serum inoculated onto confluent monolayers of Vero E6 and C6/36 (*Aedes albopictus* mosquito) cells in 6-well plastic tissue culture plates at 37°C and 28°C, respectively, in a 5% CO_2_ environment as previously described [44]. From 20–50 µL of serum were used to inoculate the cells, which were examined daily for cytopathic effect (CPE) at days 5, 7, and 14. Supernatants were harvested and two additional blind passages were performed, each passage followed by 14 days of observation for CPE. Cell culture supernatants were also monitored for evidence of viral replication by quantitative RT-PCR.

Using the remaining 100 uL of BASV-positive serum, further attempts were made to culture the virus in 5 cell lines and in suckling mice. The serum sample was split in half and diluted 1∶20 or 1∶10 in phosphate-buffered saline with 20% fetal bovine serum (FBS) to allow sufficient volume to inoculate cell cultures or mice, respectively. The first diluted sample was inoculated intracerebrally into a litter (n = 12) of 1 day old mice. Pups were observed daily for 14 days for lethality or signs of clinical illness. The second diluted sample was inoculated into 12.5 cm^2^ tissue culture flasks of Vero, BHK, LLC-MK_2_ (rhesus monkey kidney), CCL-106 (rabbit kidney) and C6/36 cells. Vertebrate cells were held at 37°C for 14 days and observed for evidence of CPE. Mosquito cells were maintained at 28°C for 10 days. Since no CPE was observed in any of the cultures, cells were subsequently fixed for transmission electron microscopy to see if viral particles could be visualized [45].

### Construction of VSVΔG-GFP Pseudotypes and BASV Serum Neutralization Testing

A pseudotype system based on a vesicular stomatitis virus (VSV) construct carrying a reporter gene for green fluorescent protein (VSVΔG-GFP) and bearing the predicted synthesized BASV glycoprotein (BASV-G) was used to generate a serum neutralization assay for BASV. Briefly, the predicted BASV glycoprotein (BASV-G) was synthesized (Genscript) and subcloned into the pCAGGS expression plasmid. Human embryonic kidney 293T cells were seeded (DMEM + 10% FBS + penicillin/streptomycin + Glutamax (Gibco) + non-essential amino acids (Gibco)) in 10 cm culture dishes 24 hours prior to transfection. Cells were transfected with 20 µg BASV-G, VSV-G, or empty pCAGGS DNA per dish following a calcium phosphate transfection protocol [46]. The culture medium was replaced 15 hours post-transfection and cells were stimulated with 6.2 mM valproic acid for 4 hours before the medium was replaced again. At 36 hours post-transfection the transfected cells were infected with VSVΔG-GFP/VSV-G pseudotypes at a multiplicity of 0.1–0.3. The inoculum was removed after 4 hours and replaced by fresh culture medium. At 24 hours post-infection, infectious supernatants were harvested, filtered through 0.45 µm filters, and concentrated 10-fold by centrifugation through a 100-kDA filter (Millipore). Concentrated viruses were aliquoted and stored at −80°C.

For serum neutralization testing, human hepatoma Huh-7 cells were seeded (DMEM +10% FBS + penicillin/streptomycin + Glutamax (Gibco) + non-essential amino acids (Gibco)) in 48-well plates 24 hours prior to infection. Per well 10 µl of pseudovirus harboring either BASV-G or VSV-G (adjusted to obtain 25–50% infection of target cells) was mixed with 10 µl of the respective serum dilution and incubated for 45 minutes at 37°C. Subsequently, the mix was added to the target cells (performed in triplicate) and cells were incubated for 24 hours at 37°C. The infected cells were detached with trypsin and washed with PBS before fixing with 2% paraformaldehyde for 1 hour at room temperature. GFP expression in infected cells was quantified by flow cytometry using a LSR II (BD Biosciences) and the collected data was analyzed with FlowJo software (TreeStar).

### Abbreviations and Nucleotide Sequence Accession Numbers

The annotated, nearly complete sequence of BASV has been submitted to GenBank (accession number JX297815). Deep sequencing reads have been submitted to the NCBI Sequence Read Archive (accession number SRA056894). Accession numbers used for the phylogenetic analyses in Figs 3, 4, and S1 are listed as follows, in alphabetical order: ABLV, Australian bat lyssavirus (NP_478343); ARAV, Aravan virus (ABV03822), BEFV, Bovine ephemeral fever virus (NP_065409); BYSMV, Barley yellow striate mosaic virus (BYSMV); CDV, Canine distemper virus (AAR32274); CHPV, Chandipura virus (ADO63669); CPV, Coastal Plains virus (ADG86364); COCV, Cocal virus (ACB47438); DURV, Durham virus (ADB88761); DUVV, Duvenhage virus (ABZ81216); EBLV1, European bat lyssavirus 1 (ABZ81166), EBLV2, European bat lyssavirus 2 (ABZ81191); EBOV, Ebola virus (AAG40171, AAA79970, BAB69010); EVEX, Eel virus European X virus (CBH20130); FDLV, Fer-de-lance virus (NP_899661); FLAV, Flanders virus (AAN73288); HeV, Hendra virus (NP_047113); HIRRV, Hirame rhabdovirus (ACO87999); HMPV, Human metapneumovirus (L_HMPVC); HPIV-1, Human parainfluenza virus type 1 (AAA69579); HPIV-2, Human parainfluenza virus type 2 (CAA40788); HPIV-3, Human parainfluenza virus type 3 (AAA46854); HPIV-4, Human parainfluenza virus type 4 (BAJ11747); INHV, Infectious hematopoietic necrosis virus (NP_042681); IRKV, Irkut virus (ABV03823); ISFV, Isfahan virus (CAH17548); KHUV, Khujand virus (ABV03824); LBV, Lagos bat virus (ABZ81171); LNYV, Lettuce necrotic yellows virus (YP_425092); MARAV, Maraba virus (AEI52253); MARV, Marburg virus (YP_001531159); MeV, Measles virus (AF266288); MMV, Maize mosaic virus (YP_052855); MOKV, Mokala virus (ABZ81206); MOUV, Moussa virus (ACZ81407); MUV, Mumps virus (AF201473); NCMV, Northern cereal mosaic virus (NP_597914); NDV, Newcastle disease virus (ADH10207); NGAV, Ngaingan virus (YP_003518294); NiV, Nipah virus (AAY43917); OVRV, Oak Vale rhabdovirus (AEJ07657); PFRV, Pike fry rhabdovirus (ACP28002); RABV, Rabies virus (NP_056797); RSV, Respiratory syncytial virus (NP_056866); RYSV, Rice yellow stunt rhabdovirus (NP_620502); SIGMAV, Sigma virus (ACU65444); SCRV, Siniperca chuatsi rhabdovirus (YP_802942); SHRV, Snakehead virus (AAD56771); SMRV, Scophthalmus maximus rhadovirus (ADU05406); SVCV, Spring viremia of carp virus (NP_116748); SYNV, Sonchus yellow net virus (NP_044286); TIBV, Tibrogargan virus (ADG86355); TUPV, Tupaia virus (AAX47602); TVCV, Tomato vein clearing virus (YP_224083); VHSV, Viral hemorrhagic septicemia virus (NP_049550); VSIV, Vesicular stomatitis virus, Indiana (NP_041716); VSNJV, Vesicular stomatitis virus, New Jersey (P16379); WCBV, West Caucasian bat virus (ABV03821); WONGV, Wongabel virus (YP_002333280).

## Supporting Information

Figure S1 **Phylogenetic analysis of the N, P, M, and G proteins of BASV and other rhabdoviruses.** Each phylogenetic tree is rooted by using the corresponding protein from human parainfluenza virus type 1 (HPIV-1), a paramyxovirus, as an outgroup. Abbreviations and accession numbers used for the phylogenetic analysis are provided in Methods.
(TIF)

Figure S2 **Confirmation of laboratory contamination by rotavirus and absence of rotavirus in BASV serum by specific PCR.** An RT-PCR assay for detection of Group A rotaviruses was performed using primers NSP3F (5′-ACCATCTWCACRTRACCCTCTATGAG-3′) and NSP3R (5′- GGTCACATAACGCCCCTATAGC-3′), which generate an 87-bp amplicon (Freeman, et al., (2008) J Med Virol 80: 1489–1496). PCR conditions for the assay were 30 min at 50°C, 15 min at 95°C for the reverse transcription step followed by 40 cycles of 95°C, 30 s/55°C, 30 s/72°C, 30 s and 72°C/7 min for the final extension. PCR products are visualized by gel electrophoresis, using a 2% agarose gel and 1 kB ladder. Rotavirus is readily detected in extracted RNA from a stool sample taken from an ongoing study of viral diarrhea in the laboratory (lane 1), but not in two separate aliquots of extracted nucleic acid from the BASV serum sample (lanes 2 and 3).
(TIF)

Table S1 **Viral reads in the deep sequencing data corresponding to the BASV-positive serum sample.**
(DOCX)

Table S2 **Demographics of 50 blood donors from Kasai-Oriental province, DRC, randomly selected for BASV antibody screening.**
(DOCX)
