# Correction: A Novel Rhabdovirus Associated with Acute Hemorrhagic Fever in Central Africa

**DOI:** 10.1371/journal.ppat.1005503

**Published:** 2016-03-18

**Authors:** Gilda Grard, Joseph N. Fair, Deanna Lee, Elizabeth Slikas, Imke Steffen, Jean-Jacques Muyembe, Taylor Sittler, Narayanan Veeraraghavan, J. Graham Ruby, Chunlin Wang, Maria Makuwa, Prime Mulembakani, Robert B. Tesh, Jonna Mazet, Anne W. Rimoin, Travis Taylor, Bradley S. Schneider, Graham Simmons, Eric Delwart, Nathan D. Wolfe, Charles Y. Chiu, Eric M. Leroy

The authors would like to correct Fig 1 and Supporting Information S2 Fig.

**Fig 1:**
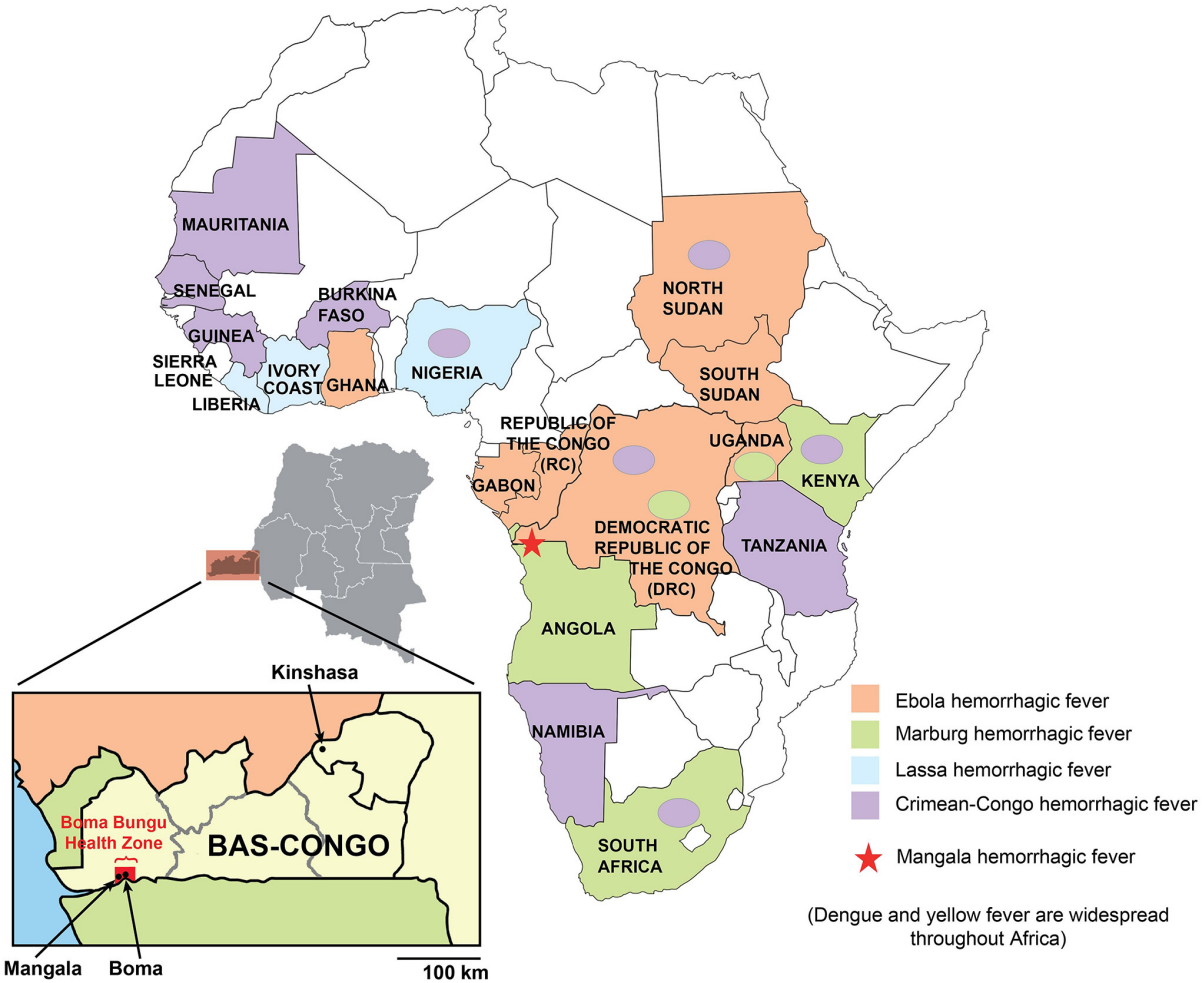
Map of Africa showing countries that are affected by viral hemorrhagic fever (VHF) outbreaks. Ebola VHF is pictured in orange, Marburg VHF in green, Crimean-Congo HF in violet, Lujo VHF in pink, and Lassa VHF in blue. Yellow fever and dengue VHF, which exhibit a wide geographic distribution throughout Sub-Saharan Africa, are not shown. Mangala village, located in the Bas-Congo province in DRC, is represented by a red star.

For Fig 1, some of the countries (Sierra Leone, Liberia, Ivory Coast, and Ghana) were mislabeled and dengue and yellow fever were not mentioned as other causes of viral hemorrhagic fever circulating in Africa. The authors now provide a corrected version of the Fig 1, with the countries correctly labelled, along with the addition of a phrase stating that dengue and yellow fever is widespread throughout Africa.

For Supporting Information S2 Fig, the original blot used to generate this figure contained additional lanes that had been removed while preparing the figure for publication. In addition, the incorrect lane 4 was inserted into the published figure. Furthermore, a black-and-white inversion and global gamma correction was applied to the entire image for ease of visualization prior to cropping. The authors now provide a corrected version of S2 Fig, with appropriate marks showing the cropped lanes that were included in the published figure, and without the inversion or gamma correction. The uncropped original blot for S2 Fig is shown as supporting information in S1 File.

The authors confirm that these changes do not alter their findings. The authors have provided raw, uncropped blots as for S2 Fig as Supporting Information S1 File.

## Supporting Information

S1 File Uncropped blots. The uncropped original blot for Supplementary S2 Fig is shown here.
(TIF)

S2 Fig Confirmation of laboratory contamination by rotavirus and absence of rotavirus in BASV serum by specific PCR. An RT-PCR assay for detection of Group A rotaviruses was performed using primers NSP3F (5′-ACCATCTWCACRTRACCCTCTATGAG-3′) and NSP3R (5′- GGTCACATAACGCCCCTATAGC-3′), which generate an 87-bp amplicon (Freeman, et al., (2008) J Med Virol 80: 1489–1496). PCR conditions for the assay were 30 min at 50°C, 15 min at 95°C for the reverse transcription step followed by 40 cycles of 95°C, 30 s/55°C, 30 s/72°C, 30 s and 72°C/7 min for the final extension. PCR products are visualized by gel electrophoresis, using a 2% agarose gel and 1 kB ladder. Rotavirus is readily detected in extracted RNA from a stool sample taken from an ongoing study of viral diarrhea in the laboratory (lane 1), but not in two separate aliquots of extracted nucleic acid from the BASV serum sample (lanes 2 and 3). Abbreviations: L = ladder, 1 = lane 1 in Supplementary Fig. 2, rotavirus diarrheal stool, RNA; 2 = lane 2 in Supplementary Fig. 2, BASV serum, RNA; 3 = lane 3 in Supplementary Fig. 2, BASV serum, cDNA (different aliquot); 4 = lane 4 in Supplementary Fig. 2, water.
(TIF)
