# Correction: Correction: A Novel Rhabdovirus Associated with Acute Hemorrhagic Fever in Central Africa

**DOI:** 10.1371/journal.ppat.1006583

**Published:** 2017-09-07

**Authors:** Gilda Grard, Joseph N. Fair, Deanna Lee, Elizabeth Slikas, Imke Steffen, Jean-Jacques Muyembe, Taylor Sittler, Narayanan Veeraraghavan, J. Graham Ruby, Chunlin Wang, Maria Makuwa, Prime Mulembakani, Robert B. Tesh, Jonna Mazet, Anne W. Rimoin, Travis Taylor, Bradley S. Schneider, Graham Simmons, Eric Delwart, Nathan D. Wolfe, Charles Y. Chiu, Eric M. Leroy

In the correction published on March 18, 2016 there were errors present in Fig 1.

**Fig 1:**
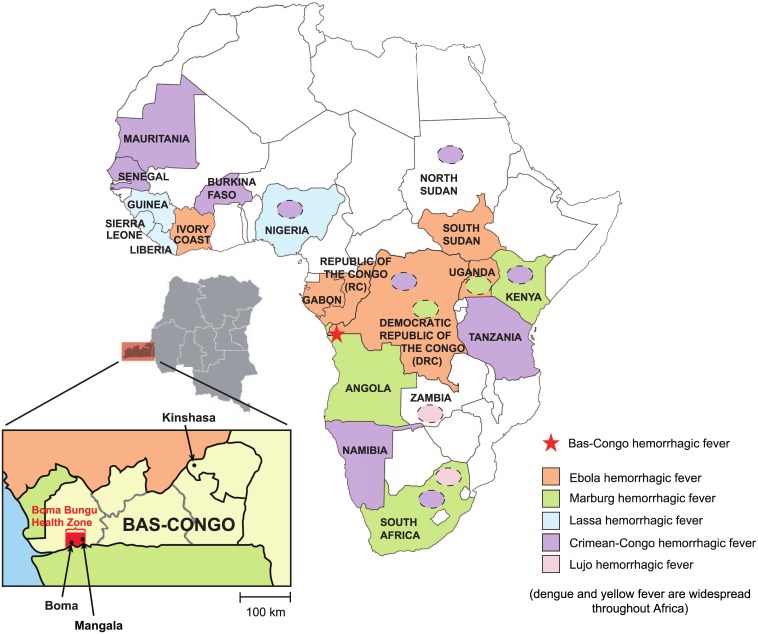
Map of Africa showing countries that are affected by viral hemorrhagic fever (VHF) outbreaks. Ebola VHF is pictured in orange, Marburg VHF in green, Crimean-Congo HF in violet, Lujo VHF in pink, and Lassa VHF in blue. Yellow fever and dengue VHF, which exhibit a wide geographic distribution throughout Sub-Saharan Africa, are not shown. Mangala village, located in the Bas-Congo province in DRC, is represented by a red star.

In the Fig 1 presented in the March 18, 2016 correction, labels were misnamed and the shading was incorrect. “Bas-Congo hemorrhagic fever” has been changed to “Mangala hemorrhagic fever” and “Lujo hemorrhagic fever” was missing from the key and the map. The location of Mangala and Boma were switched. In addition, some shading on the map was incorrect. Guinea was incorrectly labeled as being affected by Crimean-Congo hemorrhagic fever instead of Lassa hemorrhagic fever. Ivory Coast was incorrectly labeled as being affected by Lassa hemorrhagic fever instead of Ebola hemorrhagic fever. Ghana was mislabeled as having been affected by Ebola. North Sudan was also mislabeled as having been affected by Ebola. Two areas were missing labels indicating the presence of Lujo hemorrhagic fever. The authors have now corrected these errors and have provided a new corrected Fig 1. The corrected figure does not take into account changes in the epidemiology of the hemorrhagic fever viruses (e.g. the 2013 West African Ebola virus epidemic) since the time the study was published.

In addition, questions have been raised regarding the availability of additional data relevant to diagnoses of patients in Table 1. The authors indicated that no additional data relating to the field samples tested at International Centre for Medical Research at Franceville (CIRMF) are available. All relevant data was already present in the paper and Table 1, as originally published.

Further, there have been requests to verify the blot used to correct Supporting Information S2 Fig. The authors have now supplied complete annotations for the blot in order to confirm the proper labeling of the lanes. The raw, uncropped blot with all lanes annotated is available as Supporting Information S1 File. The editors are satisfied that the materials provided answer any outstanding questions about the blot.

## Supporting information

S1 File. PDF Lane 1: empty lane. Lane 2: rotavirus RNA (positive control). Lane 3: rotavirus next-generation sequencing (NGS) library (positive control). Lane 4: methicillin-resistant Staphylococcus aureus NGS library; made during period of rotavirus contamination and positive for rotavirus by NGS. Lane 5: amplified complementary DNA (cDNA) from the BASV-positive serum sample. Lane 6: DNA ladder. Lane 7: reverse-transcribed cDNA from the BASV-positive serum sample. Lane 8: methicillin-resistant Staphylococcus aureus NGS library; made after period of rotavirus contamination and negative for rotavirus by NGS. Lane 9: extracted RNA from the BASV-positive serum sample. Lane 10: water (negative control).
(PDF)
