# Supplementary figures and images for: A Novel Rhabdovirus Associated with Acute Hemorrhagic Fever in Central Africa
Source: PLoS Pathog. 2012 Sep 27;8(9):e1002924. doi: 10.1371/journal.ppat.1002924 (PMC3460624; doi:10.1371/journal.ppat.1002924)

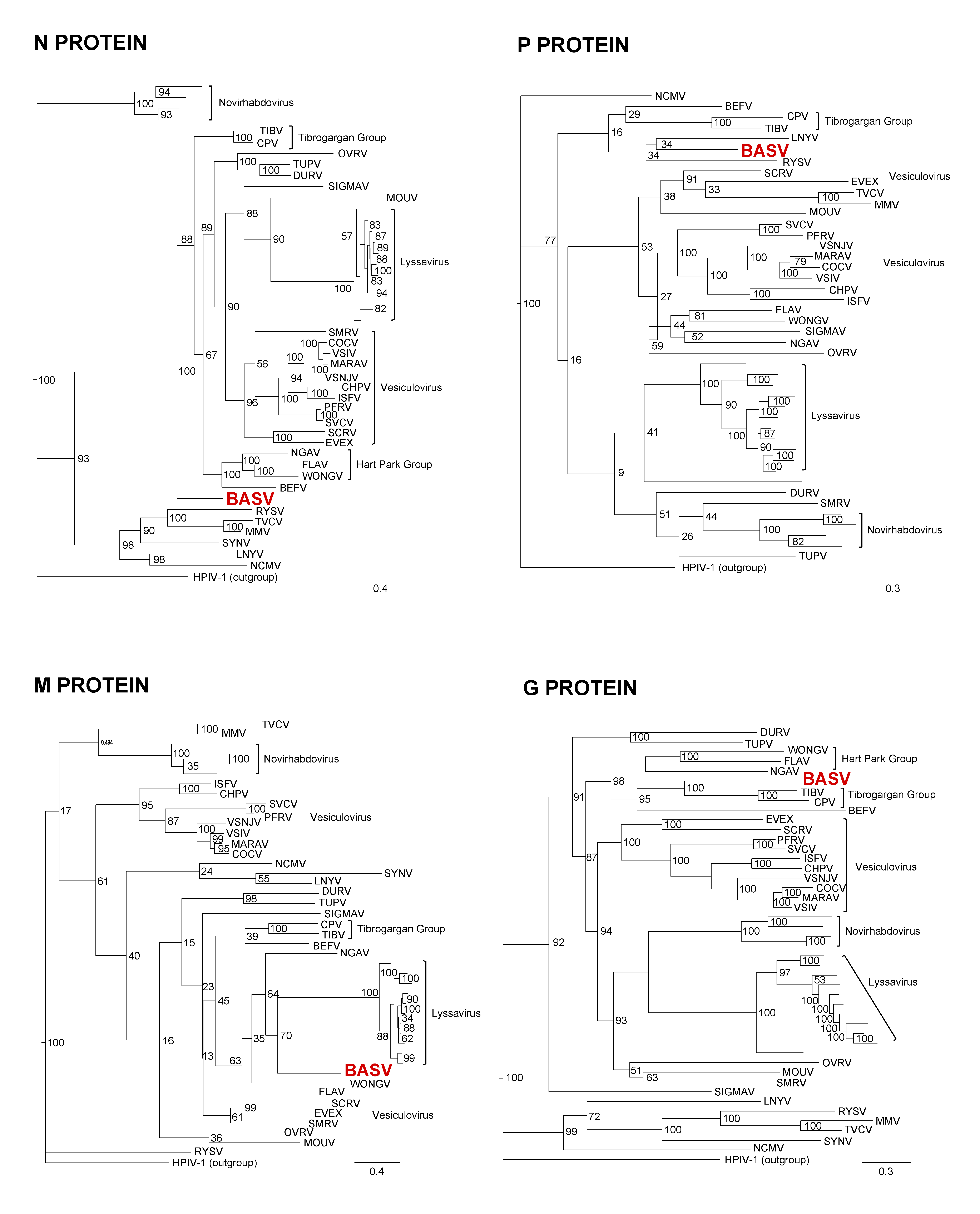

Supplement: Figure S1 — Phylogenetic analysis of the N, P, M, and G proteins of BASV and other rhabdoviruses. Each phylogenetic tree is rooted by using the corresponding protein from human parainfluenza virus type 1 (HPIV-1), a paramyxovirus, as an outgroup. Abbreviations and accession numbers used for the phylogenetic analysis are provided in Methods. (TIF) [file ppat.1002924.s001.tif]

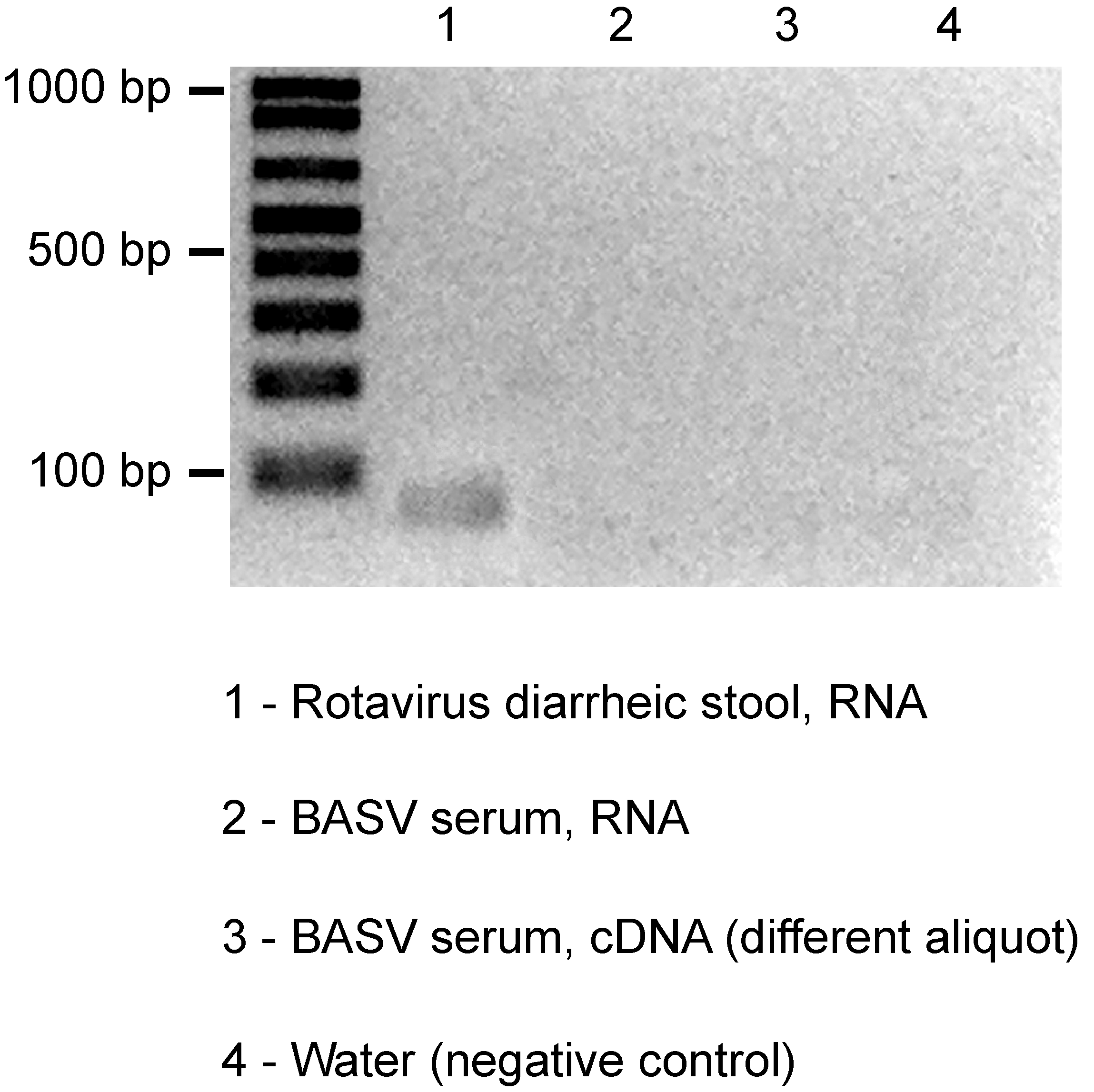

Supplement: Figure S2 — Confirmation of laboratory contamination by rotavirus and absence of rotavirus in BASV serum by specific PCR. An RT-PCR assay for detection of Group A rotaviruses was performed using primers NSP3F (5′-ACCATCTWCACRTRACCCTCTATGAG-3′) and NSP3R (5′- GGTCACATAACGCCCCTATAGC-3′), which generate an 87-bp amplicon (Freeman, et al., (2008) J Med Virol 80: 1489–1496). PCR conditions for the assay were 30 min at 50°C, 15 min at 95°C for the reverse transcription step followed by 40 cycles of 95°C, 30 s/55°C, 30 s/72°C, 30 s and 72°C/7 min for the final extension. PCR products are visualized by gel electrophoresis, using a 2% agarose gel and 1 kB ladder. Rotavirus is readily detected in extracted RNA from a stool sample taken from an ongoing study of viral diarrhea in the laboratory (lane 1), but not in two separate aliquots of extracted nucleic acid from the BASV serum sample (lanes 2 and 3). (TIF) [file ppat.1002924.s002.tif]
