# Supplementary material for: A Novel Rhabdovirus Associated with Acute Hemorrhagic Fever in Central Africa
Source: PLoS Pathog. 2012 Sep 27;8(9):e1002924. doi: 10.1371/journal.ppat.1002924 (PMC3460624; doi:10.1371/journal.ppat.1002924)
Supplement: Table S1 — Viral reads in the deep sequencing data corresponding to the BASV-positive serum sample. (DOCX) [file ppat.1002924.s003.docx]

**Table S1. Viral reads in the deep sequencing data corresponding to the BASV-positive serum sample.**

| **DETECTED VIRUS IN BASV-POSITIVE SERUM SAMPLE** | **NUMBER OF READS** | **% READS (OUT OF ~140 MILLION)** | **PRESUMED SOURCE** |
| --- | --- | --- | --- |
| BASV rhabdovirus | 29,894 | 0.021% | serum |
| Rotavirus A | 4,140* | 0.0030% | known laboratory contamination^Δ^  * |
| Tomato mosaic virus | 6 | 0.0000057% | known laboratory contamination^Δ^ |
| HERV (human endogenous retrovirus) | 10,317 | 0.0074% | human endogenous virus (in human genome) |
| MLV (murine leukemia virus), ecotropic | 717 | 0.00051% | mouse endogenous virus (mouse contamination) |
| Phage | 3,653 | 0.0026% | known laboratory contamination^¶^ |

*further analysis of the 4,140 rotavirus A reads reveal that only 73 are unique and that 98.2% of these reads are repeats, indicating that these reads are most likely due to amplicon contamination from a concurrent study of rotavirus diarrhea in the laboratory

^Δ^contamination from concurrent rotavirus diarrheal study in laboratory; confirmatory RT-PCR of two separate aliquots of extracted nucleic acid from the BASV serum sample was negative for rotavirus (Fig. S2)

^¶^from environmental bacterial contamination (*Burkholderia spp.*) in laboratory
