# Supplementary material for: A Novel Rhabdovirus Associated with Acute Hemorrhagic Fever in Central Africa
Source: PLoS Pathog. 2012 Sep 27;8(9):e1002924. doi: 10.1371/journal.ppat.1002924 (PMC3460624; doi:10.1371/journal.ppat.1002924)
Supplement: Table S2 — Demographics of 50 blood donors from Kasai-Oriental province, DRC, randomly selected for BASV antibody screening. (DOCX) [file ppat.1002924.s004.docx]

**Table S2. Demographics of 50 blood donors from Kasai-Oriental province, DRC, randomly selected for BASV antibody screening.**

| Sample Number | Material Type | Volume | Province | Health Zone | Village | Age (Years) | Sex | Ethnicity |
| --- | --- | --- | --- | --- | --- | --- | --- | --- |
| 1 | PLASMA | 100 μL | Kasai Oriental | Kole | Ekondashi | 25 | M |  |
| 2 | PLASMA | 100 μL | Kasai Oriental | Kole | Olombo Munene | 25 | F | Batetela |
| 3 | PLASMA | 100 μL | Kasai Oriental | Kole | Olombo Munene | 33 | F | Bankutshu |
| 4 | PLASMA | 100 μL | Kasai Oriental | Kole | Olombo Munene | 24 | F | Bankutshu |
| 5 | PLASMA | 100 μL | Kasai Oriental | Kole | Olombo Munene | 29 | F | Basho |
| 6 | PLASMA | 100 μL | Kasai Oriental | Kole | Olombo Munene | 18 | M | Ohindo |
| 7 | PLASMA | 100 μL | Kasai Oriental | Kole | Olombo Munene | 26 | F | Ohindo |
| 8 | PLASMA | 100 μL | Kasai Oriental | Kole | Olombo Munene | 7 | M | Ohindo |
| 9 | PLASMA | 100 μL | Kasai Oriental | Kole | Olombo Munene | 10 | M | Ohindo |
| 10 | PLASMA | 100 μL | Kasai Oriental | Kole | Olombo Munene | 7 | F | Ohindo |
| 11 | PLASMA | 100 μL | Kasai Oriental | Kole | Olombo Munene | 21 | F | Ohindo |
| 12 | PLASMA | 100 μL | Kasai Oriental | Kole | Olombo Munene | 44 | M | Ohindo |
| 13 | PLASMA | 100 μL | Kasai Oriental | Kole | Olombo Munene | 21 | F | Ohindo |
| 14 | PLASMA | 100 μL | Kasai Oriental | Kole | Olombo Munene | 15 | F | Ohindo |
| 15 | PLASMA | 100 μL | Kasai Oriental | Kole | Olombo Munene | 14 | M | Ohindo |
| 16 | PLASMA | 100 μL | Kasai Oriental | Kole | Olombo Munene | 13 | M | Ohindo |
| 17 | PLASMA | 100 μL | Kasai Oriental | Kole | Olombo Munene | 76 | M | Ohindo |
| 18 | PLASMA | 100 μL | Kasai Oriental | Kole | Olombo Munene | 16 | M | Ohindo |
| 19 | PLASMA | 100 μL | Kasai Oriental | Kole | Olombo Munene | 13 | M | Ohindo |
| 20 | PLASMA | 100 μL | Kasai Oriental | Kole | Olombo Munene | 49 | F | Ohindo |
| 21 | PLASMA | 100 μL | Kasai Oriental | Kole | Olombo Munene | 28 | F | Ohindo |
| 22 | PLASMA | 100 μL | Kasai Oriental | Kole | Olombo Munene | 12 | M | Ohindo |
| 23 | PLASMA | 100 μL | Kasai Oriental | Kole | Olombo Munene | 33 | M | Ohindo |
| 24 | PLASMA | 100 μL | Kasai Oriental | Kole | Olombo Munene | 47 | M | Ohindo |
| 25 | PLASMA | 100 μL | Kasai Oriental | Kole | Olombo Munene | 58 | F | Ohindo |
| 26 | PLASMA | 100 μL | Kasai Oriental | Kole | Olombo Munene | 27 | F | Ohindo |
| 27 | PLASMA | 100 μL | Kasai Oriental | Kole | Olombo Munene | 10 | F | Ohindo |
| 28 | PLASMA | 100 μL | Kasai Oriental | Kole | Olombo Munene | 43 | M | Ohindo |
| 29 | PLASMA | 100 μL | Kasai Oriental | Kole | Olombo Munene | 11 | M | Ohindo |
| 30 | PLASMA | 100 μL | Kasai Oriental | Kole | Olombo Munene | 12 | F | Ohindo |
| 31 | PLASMA | 100 μL | Kasai Oriental | Kole | Olombo Munene | 55 | F | Ohindo |
| 32 | PLASMA | 100 μL | Kasai Oriental | Kole | Olombo Munene | 27 | F | Ohindo |
| 33 | PLASMA | 100 μL | Kasai Oriental | Kole | Olombo Munene | 60 | F | Ohindo |
| 34 | PLASMA | 100 μL | Kasai Oriental | Kole | Olombo Munene | 75 | M | Ohindo |
| 35 | PLASMA | 100 μL | Kasai Oriental | Kole | Olombo Munene | 62 | M | Ohindo |
| 36 | PLASMA | 100 μL | Kasai Oriental | Kole | Olombo Munene | 82 | M | Ohindo |
| 37 | PLASMA | 100 μL | Kasai Oriental | Kole | Olombo Munene | 49 | M | Ohindo |
| 38 | PLASMA | 100 μL | Kasai Oriental | Kole | Olombo Munene | 42 | F | Ohindo |
| 39 | PLASMA | 100 μL | Kasai Oriental | Kole | Olombo Munene | 32 | M | Ohindo |
| 40 | PLASMA | 100 μL | Kasai Oriental | Kole | Olombo Munene | 43 | M | Ohindo |
| 41 | PLASMA | 100 μL | Kasai Oriental | Kole | Olombo Munene | 36 | M | Ohindo |
| 42 | PLASMA | 100 μL | Kasai Oriental | Kole | Olombo Munene | 32 | M | Ohindo |
| 43 | PLASMA | 100 μL | Kasai Oriental | Kole | Olombo Munene | 24 | F | Ohindo |
| 44 | PLASMA | 100 μL | Kasai Oriental | Kole | Olombo Munene | 39 | F | Ohindo |
| 45 | PLASMA | 100 μL | Kasai Oriental | Kole | Olombo Munene | 49 | M | Ohindo |
| 46 | PLASMA | 100 μL | Kasai Oriental | Kole | Olombo Munene | 25 | M | Ohindo |
| 47 | PLASMA | 100 μL | Kasai Oriental | Kole | Asenge | 49 | F | Ohindo |
| 48 | PLASMA | 100 μL | Kasai Oriental | Kole | Olombo Munene | 41 | F | BALUBA |
| 49 | PLASMA | 100 μL | Kasai Oriental | Kole | Olombo Munene | 1 | M |  |
| 50 | PLASMA | 100 μL | Kasai Oriental | Kole | Olombo Munene | 4 | F |  |
