# Supplementary material for: Correction: Correction: A Novel Rhabdovirus Associated with Acute Hemorrhagic Fever in Central Africa
Source: PLoS Pathog. 2017 Sep 7;13(9):e1006583. doi: 10.1371/journal.ppat.1006583 (PMC5589260; doi:10.1371/journal.ppat.1006583)
Supplement: S1 File. PDF — Lane 1: empty lane. Lane 2: rotavirus RNA (positive control). Lane 3: rotavirus next-generation sequencing (NGS) library (positive control). Lane 4: methicillin-resistant Staphylococcus aureus NGS library; made during period of rotavirus contamination and positive for rotavirus by NGS. Lane 5: amplified complementary DNA (cDNA) from the BASV-positive serum sample. Lane 6: DNA ladder. Lane 7: reverse-transcribed cDNA from the BASV-positive serum sample. Lane 8: methicillin-resistant Staphylococcus aureus NGS library; made after period of rotavirus contamination and negative for rotavirus by NGS. Lane 9: extracted RNA from the BASV-positive serum sample. Lane 10: water (negative control). (PDF) [file ppat.1006583.s001.pdf]

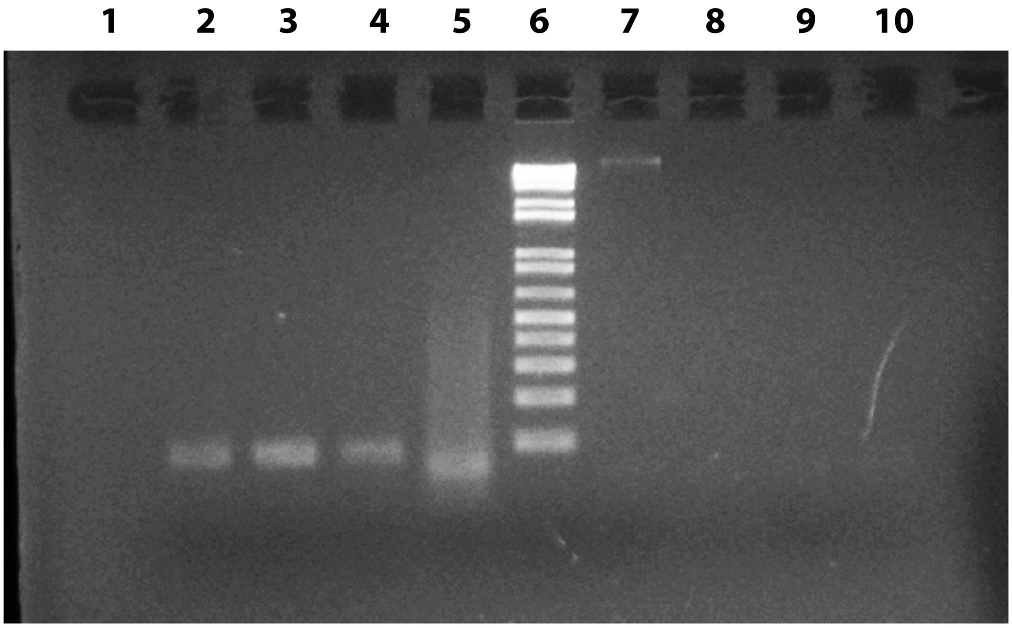

1 - empty lane

2 - rotavirus RNA (<u>positive control</u>)

3 - rotavirus next-generation sequencing (NGS) library (<u>positive control</u>)

4 - methicillin-resistant *Staphylococcus aureus* NGS library; made during period of rotavirus contamination and positive for rotavirus by NGS

5 - amplified complementary DNA (cDNA) from the BASV-positive serum sample

6 - DNA ladder

7 - reverse-transcribed cDNA from the BASV-positive serum sample

8 - methicillin-resistant *Staphylococcus aureus* NGS library; made after period of rotavirus contamination and negative for rotavirus by NGS

9 - extracted RNA from the BASV-positive serum sample

10 - water (<u>negative control</u>)
